# Effect of cholesterol‐loaded cyclodextrin treatment of bovine sperm on capacitation timing

**DOI:** 10.1111/rda.14277

**Published:** 2022-10-27

**Authors:** Gerica LaVelle, Betsy Cairo, Jennifer P. Barfield

**Affiliations:** ^1^ Department of Biomedical Sciences Colorado State University Fort Collins Colorado USA; ^2^ CryoGam Colorado Loveland Colorado USA

**Keywords:** bovine sperm, capacitation, cholesterol, cryopreservation, ionomycin

## Abstract

Pre‐loading bovine sperm with cholesterol prior to freezing is known to increase cryosurvival, though the timing of capacitation in these sperm has not been evaluated. The objective of this study was to determine if there is a potential delay in capacitation timing in these sperm due to the increased cholesterol content. Flow cytometric evaluation was utilized to assess viability, and stain technology to assess acrosome intactness (Propidium Iodide/FITC‐PNA), intracellular calcium levels (Propidium Iodide/FLUO 3‐AM) and membrane fluidity (Merocyanine 540/YO‐PRO‐1). Cholesterol‐loaded cyclodextrin (CLC) (2 mg/mL) improved post‐thaw viability to 61% from 45% in control sperm (*p* < .05). The addition of ionomycin (0.05 mM) induced capacitation in sperm by 1 h, resulting in increased intracellular calcium and increased acrosome reaction, and consequently viability loss by 3 h. Treatment with CLC significantly decreased membrane fluidity in sperm (*p* < .05). In conclusion, CLC‐treated sperm required 1 h more to capacitate when compared with non‐treated sperm based on percentage of live cells with high membrane disorder (*p* < .05). Increased cryosurvival and viability over time was observed, but longer time to capacitate may hinder fertilization capacity and/or require adjustments to timing of in vitro fertilization.

## 1 INTRODUCTION

Cryopreservation of bovine sperm facilitates the storage and transport of germplasm for use in artificial insemination (AI) and other advanced reproductive technologies. However, cryopreservation can cause irreversible cell damage, thus hindering functionality. Damage occurs to the plasma membrane during the phase transition, and can be reduced by increasing lipid composition, though this alteration also affects the ability of sperm to capacitate and the acrosome to react (Purdy & Graham, 2004a). Treating sperm with cholesterol‐loaded cyclodextrin (CLC) prior to cryopreservation increases cryosurvival rates (Mocé & Graham, 2006; Purdy & Graham, 2004a) though even with a higher number of viable sperm cells, in vitro embryo production rates and pregnancy rates from AI with control or CLC‐treated sperm were not different (Purdy & Graham, 2004b). One potential explanation of this unexpected result is a delay of frozen‐thawed CLC‐treated sperm to undergo capacitation. Our goal was to investigate the effects of CLC treatment on in vitro capacitation timing.

Cholesterol is a hydrophobic molecule that has multiple effects on membranes, including stabilizing the membrane, reducing membrane permeability, influencing the membrane phase transition, serving as a membrane antioxidant, facilitating morphological membrane characteristics, enabling cell–cell interactions, as well as providing suitable microenvironments for membrane‐associated proteins (Crockett, 1998). Cholesterol: phospholipid ratio is an important determining factor of membrane stability and fluidity at low temperature. Cholesterol maintains phospholipids in a random, lamellar arrangement as temperature decreases (Amann & Pickett, 1987) and modulates fluidity of membranes by interacting with the fatty acyl chains of phospholipids (Watson, 1981). In model membranes, increasing the cholesterol: phospholipid ratio broadens the phase transition and reduces membrane leakage and membrane phase separations (Drobnis et al., 1993). Therefore, treatment of sperm with cholesterol prior to cryopreservation could reduce sensitivity of sperm membranes to cooling damage, thus reducing or eliminating the lateral phase separation of lipids (Watson, 1981).

Cyclodextrins can be used to insert or remove cholesterol from cell membranes. Cyclodextrins are cyclic oligosaccharides obtained by the enzymatic degradation of starch, and possess an external hydrophilic face and an internal hydrophobic core (Dodziuk, 2006) that can encapsulate hydrophobic compounds, such as cholesterol (Mocé et al., 2010). These molecules have a high affinity for sterols in vitro, are very efficient in stimulating efflux of cholesterol from the membrane of sperm (e.g. Companyó et al., 2007; Visconti et al., 1999) and, if pre‐loaded with cholesterol, can insert cholesterol into cell membranes (Navratil et al., 2003). The addition of methyl residues to cyclodextrins enhance their solubility in water and ability to dissolve hydrophobic compounds (Yancey et al., 1996). Therefore, methyl‐β‐cyclodextrin (MβCD) is most commonly used to pre‐load sperm with cholesterol, creating a CLC (Mocé et al., 2010). Treating sperm with CLC prior to cryopreservation increases sperm cryosurvival rates (Mocé & Graham, 2006; Purdy & Graham, 2004a), although even with better survival no evidence has emerged that indicates this result is associated with increased fertility rates in vitro. This lack of evidence may suggest compromised sperm fertilization ability and/or the need to alter the timing of in vitro fertilization (IVF) with CLC‐treated sperm.

The objective of this study was to investigate the role of CLC on in vitro timing of capacitation in frozen‐thawed bull sperm. Assessments included viability and acrosomal integrity, intracellular calcium levels and membrane disorder. Treatment with MβCD at various concentrations, and calcium ionophore, Ionomycin, was completed to determine if there would be an effect on capacitation timing within this sperm population.

## 2 MATERIALS AND METHODS

### 2.1 Animal handling

Cattle handling and semen collection followed the National Association of Animal Breeders Management Guidelines regarding bull care. Semen was collected offsite, thus IACUC approval was not required by CSU. Rocky Mountain Sire Services has good standing with Certified Semen Services, Inc. (a subsidiary of NAAB). The 10 bovine ejaculates used for this study were provided by Select Sires, Inc. (Plain City, OH) and collected by RMSS (Bennett, CO 39°44′23° N 104°31′36° W, Climate Zone 4) from beef cattle breeds housed outside in the same facility. Collections were performed in December during breeding soundness examinations.

### 2.2 Chemicals, reagents and disposables

All research grade chemicals were purchased from Sigma Chemical Co. (St. Louis, MO). Analytical reagents such as flow cytometry fluorophores were also purchased from Sigma Chemical Co. (St. Louis, MO) and Thermo Scientific (Carlsbad, CA) unless otherwise stated. Disposables were purchased from Life Science Products (Frederick, CO) and VWR International (Radnor, PA).

### 2.3 Preparation of cyclodextrin‐loaded cholesterol

In this study, cyclodextrins were used to modify sperm membranes. The procedures of Purdy and Graham (2004a) were followed. Briefly, 1 g MβCD was dissolved in 2 mL methanol, and 200 mg cholesterol dissolved in 1 mL chloroform. The solution was mixed until clear and the solvents evaporated. The resulting powder was stored at 23°C until use, and hereafter referred to as CLC. A working solution of CLC was prepared by adding 50 mg of cholesterol‐loaded cyclodextrin to 1 mL of Tyrode’s albumin lactate pyruvate (TALP) (Graham et al., 1986) following the procedures of Purdy and Graham (2004a), and mixed thoroughly to create a CLC concentration of 50 mg/mL (345 mOsm).

### 2.4 Semen collection and cryopreservation

One ejaculate from 10 bulls was collected by artificial vagina method (Alexander, 2014). All ejaculates had a minimum concentration of 1 x 10^9^/mL, >70% normal morphology, and greater than 60% motile sperm. Ejaculates were transported to the lab immediately and then, prior to cryopreservation, each ejaculate was split into two aliquots. CLC treatment and cryopreservation of sperm were conducted as described by Mocé and Graham (2006). One aliquot served as the control, and the other was treated with 2 mg CLC/120x10^6^ sperm. Both aliquots were incubated for 15 min at 22°C, then diluted with 20% egg yolk‐Tris diluent to a concentration of 100 x 10^6^ sperm/mL and cooled to 5°C over 2 h. Once cooled, aliquots were diluted 1:1 (vol:vol) with 14% glycerolated egg yolk‐Tris diluent (final glycerol concentration 7%), loaded into 0.5 mL straws, frozen in nitrogen vapor 4 cm above the surface of liquid nitrogen for 15 min, and plunged into liquid nitrogen for storage at −196°C until thawing.

### 2.5 Flow cytometric evaluation

Cryopreserved semen from each of the 10 ejaculates was used to evaluate the effect of cyclodextrin concentration and the effect of ionomycin treatment on capacitation timing between control and CLC‐treated sperm over 180 min. For both groups (control and CLC‐treated sperm), straws were thawed in a 37°C water bath for 30 s, and motile sperm were isolated by washing thawed semen through a 45/90 Percoll® gradient (Parrish et al., 1995) at 800 g for 20 min. Supernatant was removed and sperm washed again in 2 mL TALP by centrifugation for 5 min at 300*g*. Washed sperm samples were pooled and diluted with TALP medium to 15 mL total, then separated into 2.5 mL borosilicate glass tubes suitable for analysis by flow cytometry with 2–5 x 10^6^ sperm/mL.

Within one sub‐sample group, 0.5 mL of washed, diluted sample was treated with MβCD at concentrations of 0, 1 or 2 mg/mL to test the effect of MβCD on capacitation timing by cholesterol efflux. Within a second sub‐sample group, 2 μl of 0.05 mM ionomycin was added to 0.5 mL of washed, diluted sample to act as a capacitating agent by increasing intracellular calcium levels.

Flow cytometry assessment of sperm was conducted using a BD Accuri ™ C6 flow cytometer (BD Biosciences Systems & Reagents Inc., San Jose, CA) equipped with standard optical filters FL1 533/30 nm, FL2 585/40 nm, FL3 > 670 nm, and FL4 675/25 nm operating at 66 μl/min to acquire 10,000 DNA‐positive events for each assay performed. Calibration of the instrument was performed with 19 flow cytometry calibration beads prior to use. For all flow cytometric evaluations in this study, a sperm sample killed by snap freezing was utilized as a negative control. A clean and decontamination cycle was run according to manufacturer instruction between each use. All assays below were analysed with FlowJo software (BD Biosciences Systems & Reagents Inc., San Jose, CA) programmed with gating schemes set in a logarithmic scale.

### 2.6 Fluorescent stains

Fluorescent stains were used to assess capacitation factors of spermatozoa treated with CLC. Stains included propidium iodide (PI) (0.15 mM) and FITC‐coupled peanut‐agglutinin (FITC‐PNA) (0.83 mM) to assess acrosomal integrity and viability, FLUO 3‐AM (1 mM in DMSO) and PI to assess intracellular calcium levels, and Merocyanine 540 (M540; 5 mM) with YO‐PRO™‐1 Iodide (0.1 mM) to assess membrane disorder or scrambling. Flow cytometric data on all samples were collected at 0‐, 15‐, 30‐, 60‐, 120‐, and 180 min staining times. Data were collected for all parameters on all ejaculates for both control and CLC treated sperm. Live sperm populations were considered to have compromised acrosome integrity when negative for PI and positive for FITC‐PNA (Purdy, 2008), high intracellular calcium levels with negative propidium iodide and positive FLOU‐3 AM (Nolan et al., 1992), and high plasma membrane disorder when M540 and negative for YO‐PRO™‐1 Iodide (Guthrie & Welch, 2005).

### 2.7 Statistical analysis

**FIGURE 1:**
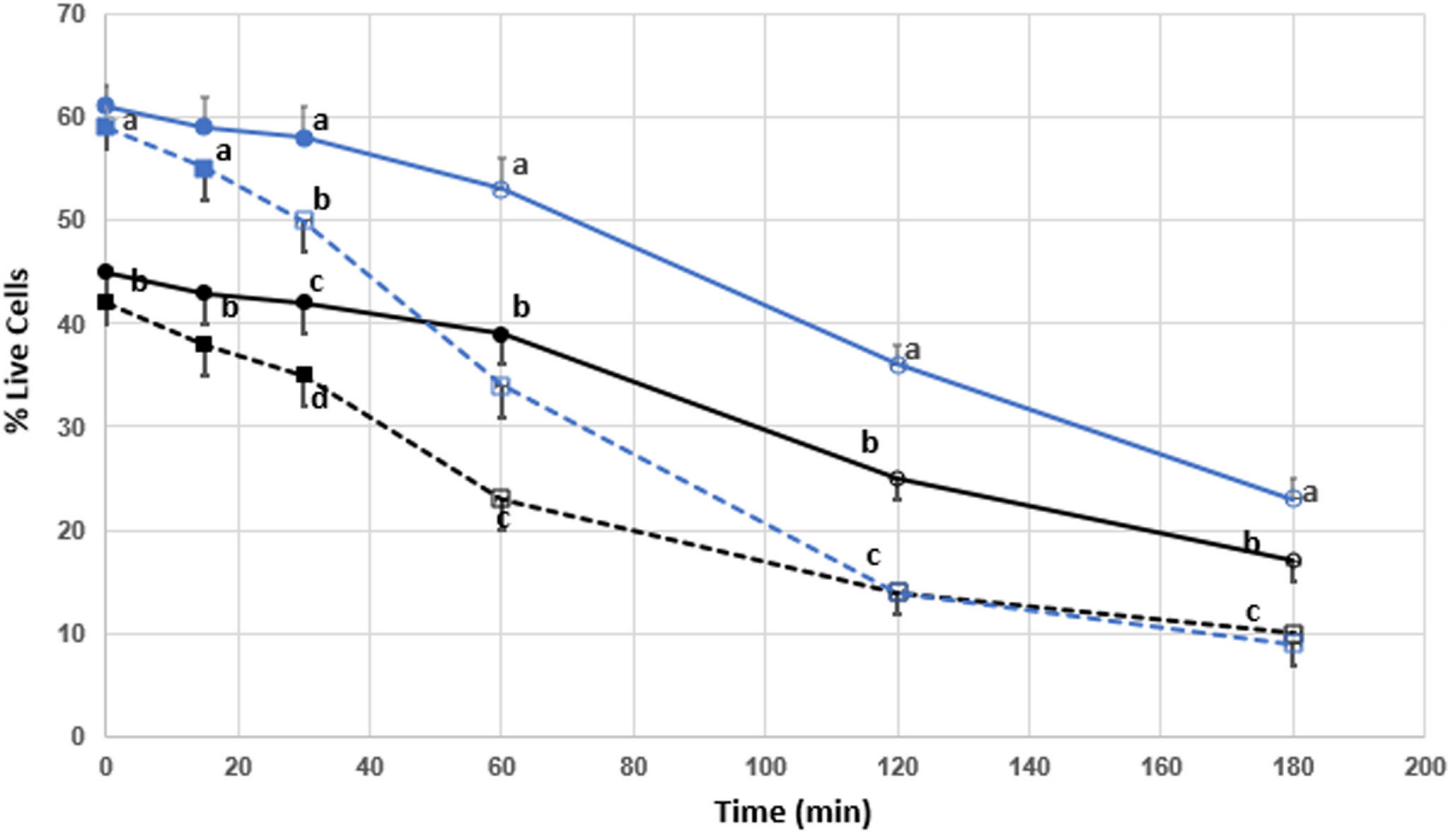
Time‐dependent post‐thaw survival of cryopreserved sperm ± CLC. percent survival of live bull sperm post‐thaw was analysed as a function of time from 0 to 180 min post‐thaw. Sperm from 10 bulls were evenly aliquoted by volume and cryopreserved without treatment (control, black solid lines) or in the presence of 2 mg/mL CLC, (blue lines) prior to freezing. Additionally, both groups were subject to treatment with 0.05 mM ionomycin to induce the acrosome reaction and capacitate sperm. Solid lines (─●─) indicate no treatment and dashed lines (‐‐‐■‐‐‐) indicate treatment with ionomycin. Open circles or squares indicate significant difference (*p* < .05) from their respective mean values at time zero. Mean values within a single time period with different superscripts indicate significant difference between treatments (*p* < .05)

**FIGURE 2:**
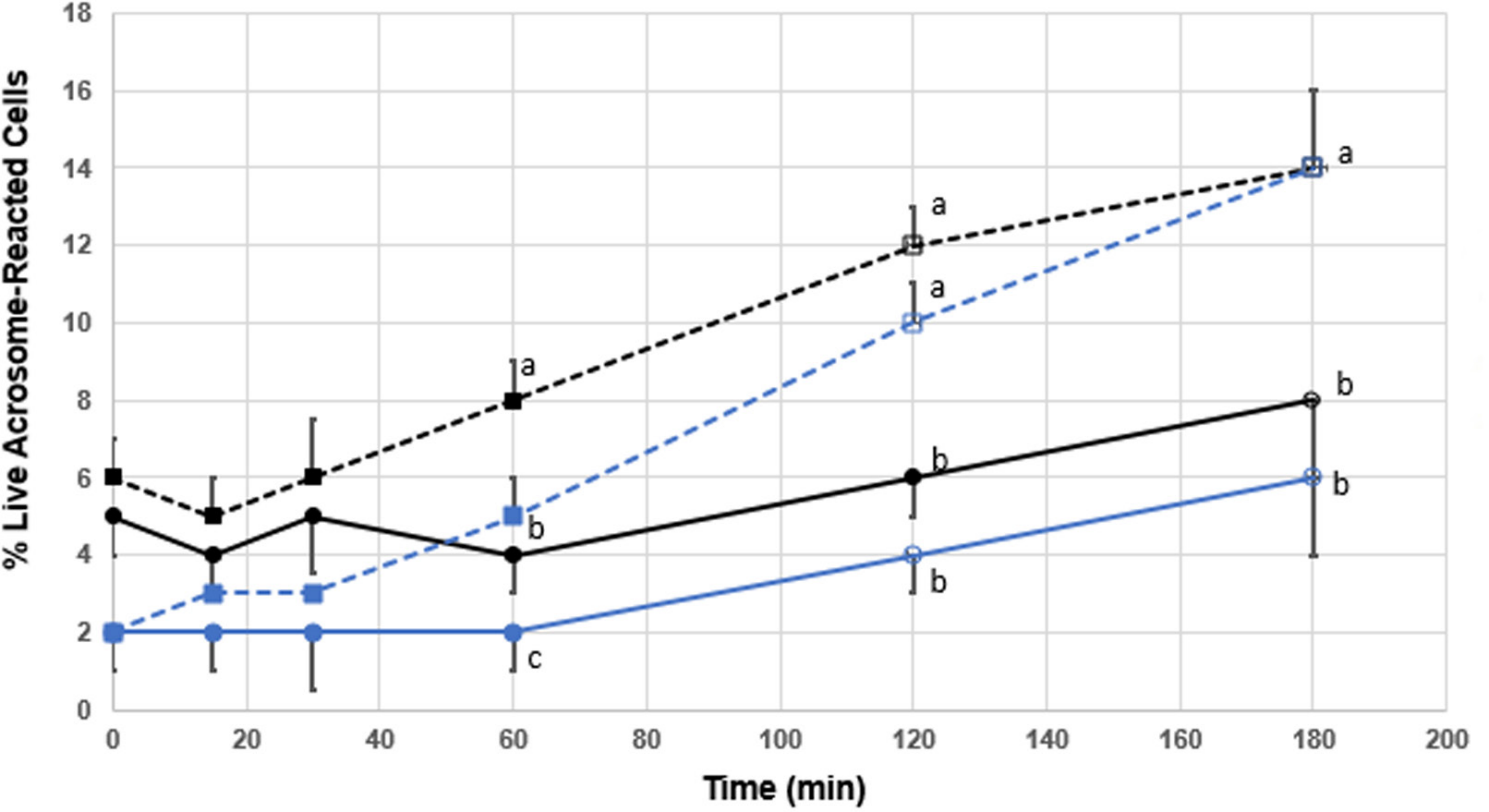
Post‐thaw survival of live acrosome‐reacted bull sperm cells ± CLC and stained with PI/FITC‐PNA analysed as a function of time from 0 to 180 min. Sperm from 10 bulls were evenly aliquoted by volume and cryopreserved without treatment (control, black solid lines) or in the presences of 2 mg/mL CLC (blue lines) prior to freezing. Additionally, both groups were subject to treatment with 0.05 mM ionomycin to induce the acrosome reaction and capacitate sperm. Solid lines (─●─) indicate no treatment and dashed lines (‐‐‐■‐‐‐) indicate treatment with ionomycin. Open circles or squares indicate significant difference (*p* < .05) from their respective mean values at time zero. Mean values within a single time period with different superscripts indicate significant difference between treatments (*p* < .05)

**FIGURE 3:**
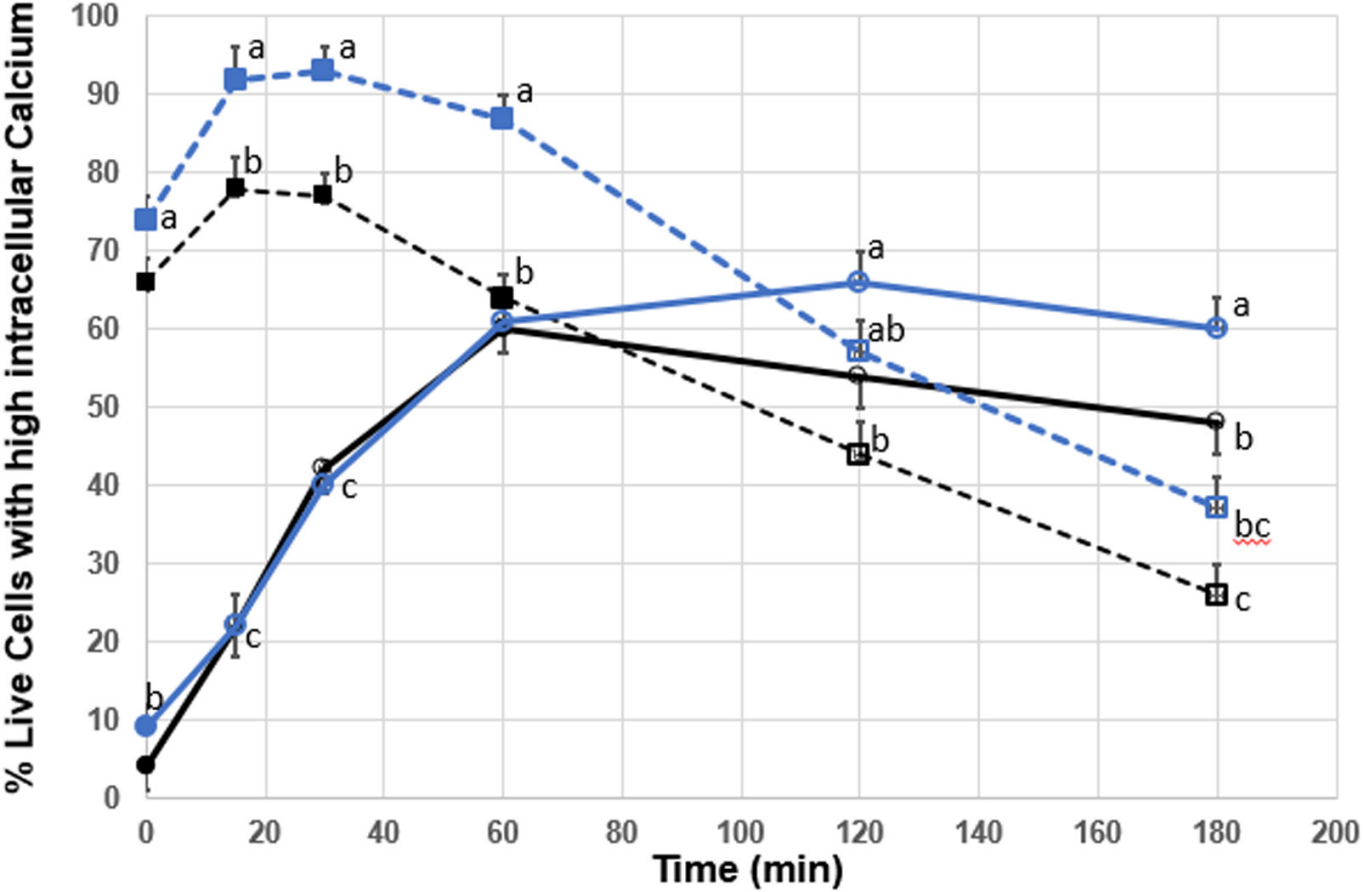
Post‐thaw survival of sperm cells incubated in the presence of CLC and revealing high intracellular calcium following exposure to staining with PI/FLUO 3‐AM analysed as a function of time from 0 to 180 min post‐thaw. Sperm from 10 bulls were evenly aliquoted by volume and cryopreserved without treatment (control, black solid lines) or in the presences of 2 mg/mL CLC (blue lines) prior to freezing. Additionally, both groups were subjected to treatment with 0.05 mM ionomycin to induce the acrosome reaction and capacitate sperm. Solid lines (─●─) indicate no treatment and dashed lines (‐‐‐■‐‐‐) indicate treatment with ionomycin. Open circles or squares indicate significant difference (*p* < .05) from their respective mean values at time zero. Mean values within a single time period with different superscripts indicate significant difference between treatments (*p* < .05)

**FIGURE 4:**
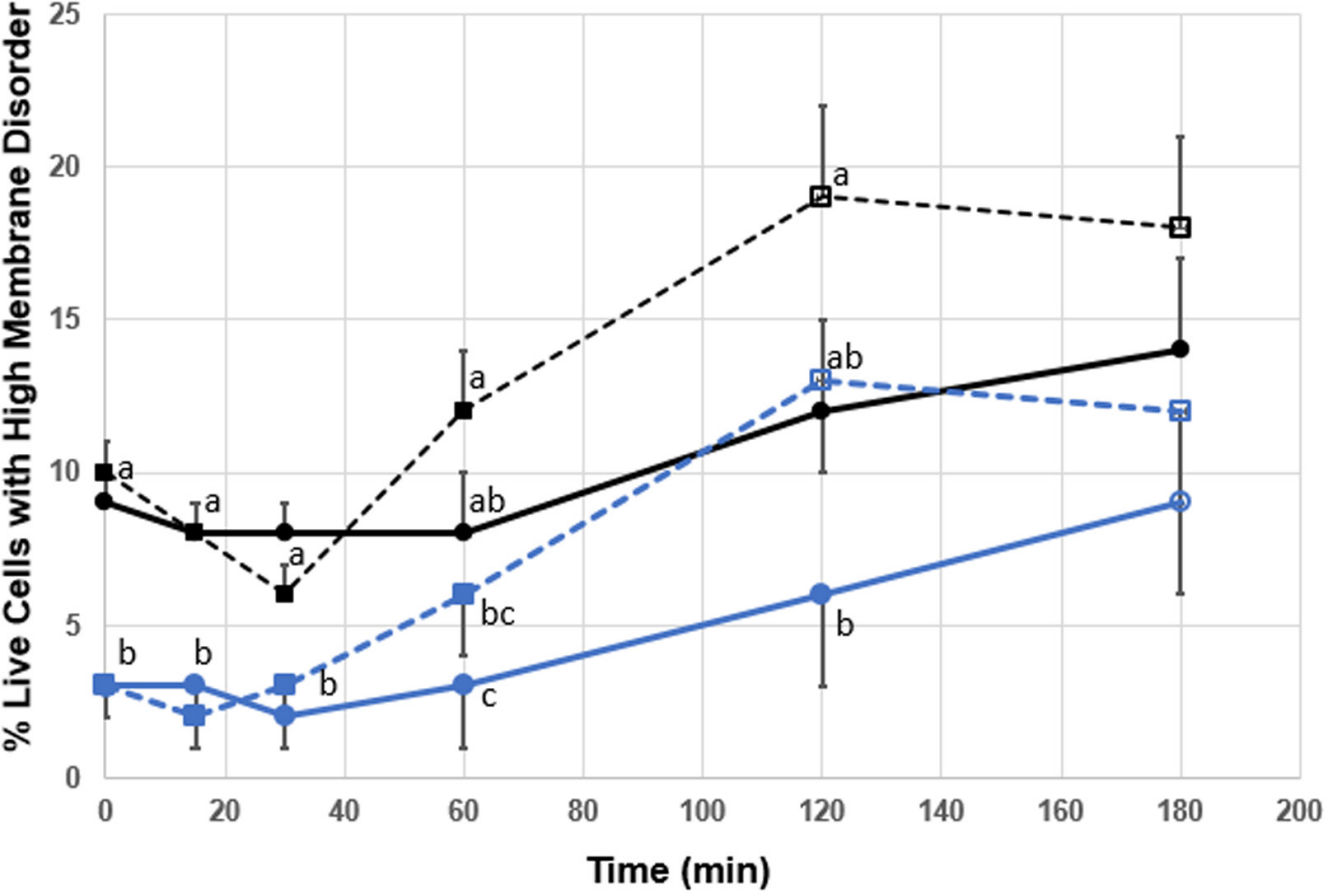
Membrane disorder in live post‐cryo‐preserved sperm cells incubated in CLC. The degree of disorder was revealed by the application of the stain merocyanine 540/YO‐PRO‐1 with cells presenting as high fluidity by percent of live bull sperm post‐thaw and analysed as a function of time from 0 to 180 min post‐thaw. Sperm from 10 bulls were evenly aliquoted by volume and cryopreserved without treatment (control, black solid lines) or in the presences of 2 mg/mL CLC (blue lines) prior to freezing. Additionally, both groups were subject to treatment with 0.05 mM ionomycin to induce the acrosome reaction and capacitate sperm. Solid lines (─●─) indicate no treatment and dashed lines (‐‐‐■‐‐‐) indicate treatment with ionomycin. Open circles or squares indicate significant difference (*p* < .05) from their respective mean values at time zero. Mean values within a single time period with different superscripts indicate significant difference between treatments (*p* < .05)

All statistical analyses were performed with SAS software (version 9.4; SAS Institute Inc., Cary, NC, USA). Separate analyses were performed for the percent live sperm, live acrosome‐reacted sperm, live sperm with high intracellular calcium levels, and live sperm with high membrane fluidity. Values for these parameters are presented as the mean ± SEM (Figures 1, 2, 3, 4). Percentage data were transformed (arcsin) prior to analysis. Initially, MβCD levels (0, 1and 2 mg/mL) were analysed by ANOVA, at each time point within each treatment (control, control+ionomycin, CLC and CLC + ionomycin) to determine if MβCD administration affected sperm parameters. No differences (*p* > .05) were determined in any sperm parameter for the different MβCD levels within the four sperm treatments at any specific time point. Therefore, data for the three MβCD treatments were pooled in the final analyses. A Repeated Measurements Analysis of Variance was used to detect differences temporally within each treatment. Within each time point, treatment differences were determined using ANOVA and means were separated using Student–Newman–Keuls (SNK).

## 3 RESULTS

From the 10 ejaculates used in this split ejaculate study, there are four treatment groups (control, control + ionomycin, CLC and CLC + ionomycin) which were evaluated for acrosomal integrity, intracellular calcium level and membrane disorder by flow cytometry over time up to 180 min. The treatment of CLC prior to cryopreservation was the main effect, with ionomycin as a secondary treatment being tested. Varied M*β*CD concentration levels of 0, 1 and 2 mg/mL revealed no difference at any time points (Table 1).

**TABLE 1:** Average percentage of live cells observed between time 0 and 180 min across treatments concentrations in mg/mL. No significant differences were found (*p* > .05)

Control				CLC		
Time (min)	[0]	[1]	[2]	[0]	[1]	[2]
0	44.8	44.7	44.9	61.2	61	61.1
15	43.1	42.8	42.5	59.8	59.3	58
30	42	42.3	41.8	58.2	58.1	56.7
60	39.4	39.9	38.6	52.6	54.6	52.7
120	24	25.9	24	33.5	36.9	37.1
180	16.3	17.2	16	22.1	23.4	22.2

Control + Ionomycin				CLC + Ionomycin		
Time (min)	[0]	[1]	[2]	[0]	[1]	[2]
0	42.8	41.9	41.7	60.4	59.3	58.1
15	38.7	38.5	37.6	56.7	54.8	54.6
30	35.9	35.1	32.6	54.1	48.7	47.6
60	24.5	24.1	21.3	40.1	33.5	30.8
120	14.4	14.3	12.4	15.4	14.5	11.3
180	10.1	10.6	9.2	10.7	9.3	7.7

The percentage of live post‐thaw sperm cells treated with CLC prior to freezing exhibited significantly higher viability compared with control sperm at time zero (*p* < .01), and displayed the highest percent of live cells at all time points between 0 and 180 min (Figure 1). From time 0, significant viability loss was seen at 30 min for ionomycin‐treated CLC sperm, at 60 min for ionomycin‐treated control sperm and CLC sperm, though not until 120 min for control sperm (*p* < .05). The greatest loss of cell viability (50 percentage points) was seen in the CLC sperm treated with ionomycin though this group displayed the second highest post‐thaw viability at time 0. The least amount of viability loss was seen in the control sperm without ionomycin treatment with 28 percentage points lost at 3 h.

A significantly higher percentage of live acrosome‐reacted sperm was noted in the non‐treated CLC group, treated control group, and treated CLC sperm group at 120 min as compared to time 0 for each treatment (*p* < .05). A significant difference was not seen in control sperm until 180 min (*p* < .05). CLC sperm displayed the lowest percent of live acrosome‐reacted cells at all time points (Figure 2). The highest increase in percent of live acrosome‐reacted cells was seen in CLC sperm and control sperm treated with ionomycin (12 and 8 percent increase respectively).

Ionomycin treatment resulted in sperm intracellular calcium increasing quickly, peaking at 15 and 30 min for control and CLC sperm respectively, with non‐treated control sperm peaking at 60 min post‐thaw and at 120 min for CLC sperm. Ionomycin treatment revealed a decline in percentage of live cells with high intracellular calcium after peaking at time 30 for both control and CLC sperm, indicating that the cells had capacitated. When comparing peak percentage of live cells with high intracellular calcium to the final time point of 3 h, CLC sperm treated with ionomycin had the greatest drop in percentage points (56) and CLC sperm with no treatment had the least amount of decline at 6 percent. A significant difference was determined in non‐treated control and CLC cells at 15 min and 120 min for ionomycin‐treated groups (*p* < .05). These results validate the use of ionomycin to induce capacitation by increasing intracellular calcium levels (Figure 3).

When analysing percentage of live cells exhibiting membrane disorder, there was no significant difference in control sperm, and CLC sperm only displayed significant difference at 180 min (*p* < .05). Ionomycin‐treated control and CLC sperm were both different at 120 min from time 0 (*p* < .05). Non‐treated CLC sperm displayed the lowest percentage of live cells exhibiting membrane disorder (Figure 4). Similar percentage difference between the lowest point to peak was matched between non‐treated control and CLC sperm (6 and 7 percent respectively) and between ionomycin treated control and CLC sperm (13 and 11 percent respectively). These similarities indicate that increased cholesterol within the plasma membrane of the sperm cells limited disorder potential, and that when treated with ionomycin displayed similar reaction results across the control and CLC sperm.

## 4 DISCUSSION

Cryopreservation of bovine sperm negatively effects viability, induces synthesis of reactive oxygen species, and causes DNA damage (Gürler et al., 2016). In this and previous studies, treatment of bovine sperm with CLC prior to freezing supports increased post‐thaw viability both initially after thaw and over time (Mocé & Graham, 2006; Purdy & Graham, 2004a). Our results displayed significantly higher percentage of live post‐thaw sperm cells treated with CLC prior to freezing compared with control sperm at time zero (*p* < .01). These improved cryosurvival rates suggest that CLC‐treated sperm can more effectively withstand the damaging conditions of cryopreservation, and yield otherwise unlikely survivability. Recent studies have indicated increased post thaw viability when CLC is added prior to cryopreservation of bovine sperm packaged for low dose insemination, which is a potential application for the use of CLC to optimize the preservation of valuable genetic donors (Lone et al., 2021).

In previous studies, M*β*CD improved the capacitation status as assessed by the increase in plasma membrane fluidity, intracellular calcium concentration, induced acrosome reactivity and zona pellucida (ZP)‐binding ability (Águila et al., 2015), and increased rates of activation and fertilization (Kato et al., 2011) in bovine sperm in vitro. However, our results indicated no significant difference (*p* > .05) at 0, 1 and 2 mg/mL at all time points. This difference may indicate that the concentration of M*β*CD used in our present study was not high enough to induce cholesterol efflux in CLC‐treated sperm, as in previous studies there was a decrease observed in a dose‐dependent manner compared to the control sperm (Águila et al., 2015). High concentrations of M*β*CD were not used due to indication of DNA integrity being compromised at 5 mM (Águila et al., 2015), however, treatment at these levels may be necessary to see an effect on CLC‐treated sperm. CLC‐treated sperm are estimated to have an increased cholesterol: phospholipid ratio from 0.45 to 0.9 or higher (Purdy & Graham, 2004a), therefore, to see an effect of M*β*CD on these sperm, treatment with up to twice the concentration used in the current study may be required. Further studies assessing M*β*CD effect at concentrations greater than 2 mg/mL would be required to evaluate cholesterol efflux in CLC‐treated sperm, while also assessing potential damage to DNA integrity.

When assessing percent of live acrosome‐reacted sperm, the group treated with CLC was associated with the lowest percent of live cells reacted at all time points, supporting the hypothesis that CLC‐treated sperm require more time to react acrosomally, and therefore capacitate more slowly compared with control sperm. Previous studies evaluated this reaction over 30 min (Purdy & Graham, 2004b), however evaluation over a longer 3 h timeframe allowed for a more in depth assessment. This hypothesis is also supported when assessing percent of live cells exhibiting membrane disorder. Our results reinforce the inference that adding cholesterol to frozen‐thawed sperm cells cultured in the above conditions induces membranes to become more rigid (Purdy et al., 2005), an observation based on the finding of increased membrane stability of CLC‐treated sperm. This was true for sperm cells both treated with ionomycin and non‐treated CLC sperm, with significant difference seen between most time points (*p* < .05).

Ionomycin, a potent and selective calcium ionophore, has been used as a research tool to stimulate hyperactivation in bovine sperm. A similar calcium ionophore A23187 has been used to induce capacitation of bovine sperm (Purdy & Graham, 2004b), although A23187 acts by incorporating into the sperm plasma membrane and transporting calcium stores across the plasma membrane (Talbot et al., 1976), whereas Ionomycin acts by inducing the release of cytosolic calcium stores (Morgan & Jacob, 1994). Our results support the work of Purdy and Graham (2004b) with CLC‐treated sperm and control sperm exhibiting similar percentages of cells with high intracellular calcium. Figure 3 displays a sharp increase in the percentage of live cells with increased intracellular Ca^2+^ indicating capacitation was occurring, though after 60 min (con) or 120 min (CLC‐treated) there is a steady decline in live cells displaying high intracellular calcium, suggesting that population of cells died once capacitated. Sperm treated with ionomycin displayed the highest increase in percentage of live acrosome‐reacted cells over time, also supporting the notion that capacitation has occurred. Loss of motility was also observed in subsamples treated with ionomycin. In total, these results support other reports, in addition to providing insight on the effect of ionomycin treatment on bovine sperm capacitation when accessing acrosome reaction, intracellular Ca2+ levels, and membrane disorder. These results support the hypothesis that the additional cholesterol added to sperm via CLC‐treatment does delay capacitation timing in vitro, as shown from later acrosome reaction, decreased membrane disorder, and effect on intracellular calcium levels.

Cholesterol loading prior to freezing resulted in delayed capacitation timing. This delay may be the cause for the absence of increased fertility in vitro, but further in vitro investigation is required for confirmation of this conclusion. Additionally, further investigation of the molecular mechanisms involved in delayed capacitation of CLC‐treated sperm would help determine the cause of the delay and determine why there is no improvement in fertility associated with CLC‐treated sperm. Paired with additional study of potential uses for CLC treatment in cryopreservation of low‐dose insemination units, CLC treatment could highly benefit the cryopreservation of valuable genetic sires and/or provide higher yield of insemination units per ejaculate.

## 5 CONCLUSION

In conclusion, treatment of bovine sperm pre‐loaded with cholesterol prior to freezing via CLC (2 mg/mL) improved cryosurvival and post‐thaw viability, initially and over time by significantly decreasing membrane fluidity in sperm. However, CLC‐treated sperm exhibit delayed capacitation by 1 h based on assessment of viability and acrosomal integrity, intracellular calcium levels, and membrane disorder. MβCD treatment (1 mg/mL and 2 mg/mL) did not impact capacitation, though ionomycin (0.05 mM) induced capacitation by 1 h, resulting in increased intracellular calcium and acrosome reaction. Further investigation of retained cholesterol post‐thaw is required, as longer time to capacitate may hinder fertilization capacity and/or require adjustments to IVF timing.

## AUTHOR CONTRIBUTIONS

GL and BC conceived and designed the study. GL performed the flow cytometric assay, statistical analyses and drafted the manuscript. All authors edited, revised and accepted the article.

## FUNDING INFORMATION

This work was privately funded by CryoGam Colorado, LLC.

## CONFLICT OF INTEREST

The authors declare no conflict of interest.

## Data Availability

The data that support the findings of this study are available from the corresponding author upon reasonable request.
